# A Review of Recent Advances in Our Understanding of Neisseria gonorrhoeae

**DOI:** 10.7759/cureus.43464

**Published:** 2023-08-14

**Authors:** Kajal Mahapure, Akhilesh Singh

**Affiliations:** 1 Accident and Emergency, Jawaharlal Nehru Medical Collage, Datta Meghe Institute of Higher Education and Research, Wardha, IND; 2 Emergency Medicine, Jawaharlal Nehru Medical Collage, Datta Meghe Institute of Higher Education and Research, Wardha, IND

**Keywords:** health, microorganisms, disease, infection, treatment

## Abstract

Gonorrhoea is an infection caused by bacteria that has recently been detected in humans and typically spreads through sexual contact. It leads to significant health issues in both prosperous and impoverished countries, culminating in significant yearly expenditures for diagnosis and treatment. Young adults who are involved in unprotected sexual activity and are promiscuous are particularly susceptible to gonorrhoea. It has been estimated that approximately 86.95 million individuals globally acquire the virus each year. Gonorrhoea has been reported to affect a variety of body parts, including the cervix in women and the urethra in males, as well as other areas such as the eyes, anus, throat, and, on rare occasions, the joints. It is momentarily the second most frequently reported sexually transmitted disease (STD) by the Centers for Disease Control and Prevention (CDC), trailing only chlamydia. Since the early 2000s, gonorrhoea cases have been on the rise globally, especially across many European nations, with an elevated prevalence among populations at higher risk of getting sexually transmitted infections (STIs), such as men who have sex with men and young heterosexual individuals. The fundamental objectives of gonorrhoea management techniques are to prevent, identify, and cure infections in patients and their partners in addition to minimizing the disease's stigma. It additionally involves monitoring antibiotic resistance and treatment failures, and it also involves advocating appropriate antimicrobial medication usage and stewardship.

## Introduction and background

Gonorrhoea, which is reported to be caused by the bacterium *Gonococcus*, is a serious global health issue resulting from sexually transmitted infections (STIs). The bacterium is known to infect the lining of the urinary and genital organs but has also been found to adhere to the outer lining of the cervix, which mainly comprises flat cells [1,2]. Transmission of the bacteria has been reportedly found to take place during coitus through direct contact with mucous membranes in the oropharynx, anal canal, urinogenital tract, and eyes (conjunctivitis). This often results in cervicitis in females and urethritis in males. Most males who suffer from symptomatic gonococcal urethritis, post-medical treatment, are no longer found to be contagious. However, the presence of untreatable gonorrhoea may increase the incidence of complications due to the infection. With over 78 million annual cases worldwide and limited treatment options in low-income nations and marginalised groups in wealthier countries [3-5].

Although *Neisseria gonorrhoeae* may also colonise the anal mucosa, nasopharyngeal, and ocular areas, its primary colonisation site is found to be the vaginal mucosa [6]. The adverse outcomes mainly arise from the damage caused by the activation of the innate immune response at the colonisation sites since *N. gonorrhoeae* is not known to produce any potent exotoxins. In females, untreated infections of the ascending genital tract may lead to complications such as ectopic pregnancy, pelvic inflammatory illness, and infertility [7]. Transmitting these bacteria from mothers to their unborn offspring may result in neonatal blindness [8]. Untreated *N. gonorrhoeae* may lead to disseminated gonococcal infection, which is known to cause infectious arthritis and endocarditis [9]. In some cases, *N. gonorrhoeae* may penetrate the circulation and disseminate, leading to cutaneous and tendon or joint infections and, rarely, endocarditis or meningitis [10].

During childbirth, infected women may pass on the gonococcal infection to their babies, resulting in eye-related conditions (ophthalmia neonatorum) or, in rare instances, widespread illness. Asymptomatic pharyngeal or rectal gonorrhoea is also common. Moreover, gonococcal infection has been found to increase the transmission and acquisition of HIV [11].

Gonorrhoea is still a significant threat to global public health, with the World Health Organisation (WHO) estimating 78.31 million new cases among people aged 15 to 49 in 2008 [12]. In accordance to the Centres for Disease Control and Prevention (CDC), gonorrhoea is the second most prevalent bacterial disease in the United States [13]. In 2015, around 395,200 cases of gonorrhoea were reported, marking a 27% increase from 2012. However, the actual number of cases is likely higher, especially in resource-limited areas where the lack of sensitive diagnostics or testing centres and inadequate case reporting lead to under-recognition and under-reporting [13].

## Review

Methodology

**Figure 1:**
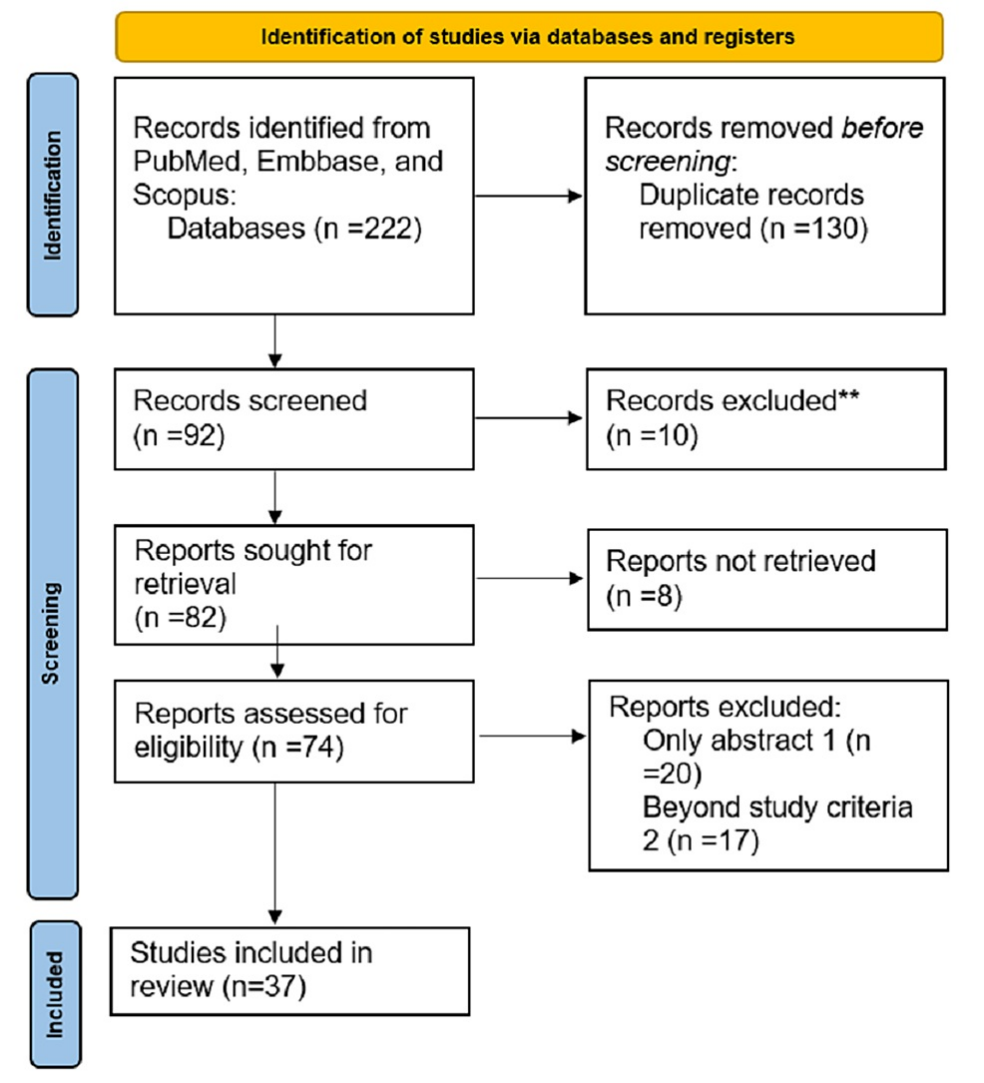
PRISMA 2022 flow diagram depicting our study selection process PRISMA: Preferred Reporting Items for Systematic Reviews and Meta-Analyses

The authors conducted a literature search using the following electronic search keywords: "population" with search fields "children," "pediatric," and "child," and "Alcohol" and "Road Traffic accident." The search was performed in worldwide reputed search engines such as PubMed, Scopus, Web of Science, Embase, and Ovid/Medical Literature Analysis and Retrieval System Online (MEDLINE) databases, typically involving English language literature. The search fields were adjusted and alternated until no new articles were uncovered. The search was deemed to be completed once no further relevant articles were found (Figure 1).

Microbiology

Neisseria species are gram-negative diplococci. They are generally found in the mucosal membranes of reptiles and mammals. Some of them are also causative of primary human infections. These cocci reportedly have a 0.6-1 μm diameter and usually occur in pairs with flattened adjoining sides. One of the exceptions among them is *Neisseria elongata*, which consists of approximately 0.5 μm wide small rods, typically organised in short chains or diplobacilli [14]. All microorganisms in the *Neisseriaceae *family are found to be aerobic chemoorganotrophs with a genomic DNA content of 46.50-53.50 mole per cent cytosine + guanosine. They tend to exhibit various levels of natural transformation efficiency with homologous DNA and have specific nutritional requirements, thriving at temperatures between 35 to 37 degrees Celsius. Some Neisseria species are reported to be a-hemolytic, while others produce a yellowish-green carotenoid pigment and have capsules [15].

Antigenic structures

The cytoplasmic membrane of *N. gonorrhoeae* and *Neisseria meningitidis*, like other gram-negative bacteria, is generally found to be separated from the outermost membrane by the periplasmic space. The outer leaflet of this membrane serves a crucial role in interactions with the host and can either facilitate host infection or serve as a target for the host's immune response. This outer leaflet comprises a complex combination of phospholipids, lipooligosaccharides, and proteins.

Pili

Pili are reportedly observed to have hair-like appendages that tend to branch out to several micrometres from the surface of bacteria. Pili can be broken down into two types: virulent T1-T2 and avirulent T3-T5. These structures help predominantly aid in genetic material transfer across strains, conformity to human mucosal cell surfaces, host cell penetration, and phagocytosis avoidance. The genetic discrepancy in pilus form between T1 and T5 enables the organism to manipulate its antigenic structure, avoiding identification by host immune cells.

Porin proteins (Por proteins)

Por proteins are typically known to extend from the cell membrane surface of gonococcal bacteria, and then they form pores that allow nutrients to enter the cell. These proteins may possess an influential power in the intracellular survival of gonococci in neutrophils by blocking phagosome-lysosome fusion. Furthermore, the interaction of the Por protein with complement elements C3b and C4b has been reportedly found to determine the gonococci's resistance to being killed by normal human blood.

Opacity proteins (Opa proteins)

Opa proteins are known to play a significant role in the attachment of gonococci to host cell receptors, such as CD66 or carcinoembryonic antigen-related cell adhesion molecules, facilitating adherence to host cells.

Reduction of modifiable protein (RMP protein)

This protein is antigenically conserved in all gonococci and collaborates with Por to create pores on the cell surface, preventing host IgG from having a bactericidal effect.

Lipooligosaccharide (LOS)

The lipooligosaccharide is known as gonococcal lipopolysaccharide (LPS). It is generally devoid of elongated O-antigen side chains. The endotoxic effects of LOS are primarily responsible for the toxicity observed in gonococcal infections. Outer cell membrane proteins I-III, lipooligosaccharide (endotoxin), and capsule are known to play critical roles in antigenic variation and the induction of an inflammatory response.

Other proteins

Apart from Opa and Lip (H8), a surface-exposed protein called GBP (ferric-binding protein) generally manifests when accessible iron resources are limited, as is the case during human infections. Also, gonococci are reportedly known to produce an IgA1 protease, breaking down and inactivating the significant human mucosal immunoglobulin IgA1 [16].

Epidemiology

*N. gonorrhoeae* poses a significant public health challenge and has a record of being the second most prevalent bacterial sexually transmitted disease worldwide [17]. The WHO has provided an estimation of the annual diagnosis of approximately 106 million new cases in adults, with many additional infections likely going undetected [18]. In the United States, *N. gonorrhoeae* has been found to be the second most commonly reported sexually transmitted disease, with over five lakh cases recorded each year [19]. Gonorrhoea infection has been reportedly found to incline towards a modest male predominance due to the higher likelihood of males experiencing urogenital symptoms, and there is an increasing detection rate among males who have sex with males. Over the past decade, the prevalence of gonorrheal sexually transmitted diseases has been exacerbated by the emergence of antibiotic-resistant strains [20].

Gonorrhoea exclusively affects humans, and no animal reservoirs have reportedly been found to be in existence for the disease. The infection source is primarily asymptomatic female carriers, although less frequently, symptomatic patients can also contribute to transmission. The condition is predominantly transmitted through sexual contact (venereal), with transmission from men to females being more efficient than in the reverse direction. Additionally, during childbirth, there is a risk of transmission from mother to baby.

Clinical manifestations of *N. gonorrhoeae*

Gonococci primarily have been found to colonise the rectum or mucous membrane of the genitourinary tract. Infections caused by these organisms can lead to tissue invasion, persistent inflammation, fibrosis, or localised infections with pus formation. A higher percentage of females compared to males have been found to be asymptomatic carriers, serving as a reservoir for sustaining and spreading gonococcal diseases.

Urinogenital tract infections usually present with yellow, purulent urethral discharge and painful urination in men. In females, the infection can spread from the endocervix to the urethra and vagina, causing greenish-yellow cervical discharge, often accompanied by inter-menstrual bleeding. Males may also experience acute purulent urethritis, prostatitis, and epididymitis, while females can develop acute cervicitis. Progression of the infection to the uterus may lead to fibrosis, salpingitis (inflammation of the fallopian tubes), and pelvic inflammatory disease (PID). PID, also known as Fitz-Hugh-Curtis syndrome, can result in perihepatitis, ectopic pregnancy, or infertility.

Rectal infections are reportedly common in males who engage in sexual activity with other men and are characterised by constipation, painful defecation, and purulent discharge. Pharyngitis has been found to occur due to oral-genital contact, and individuals with pharyngitis may exhibit purulent pharyngeal exudate. Ophthalmia neonatorum is transmitted to newborns during childbirth when they come into contact with their mothers' infected birth canals. If left untreated, acute conjunctivitis can lead to blindness.

Disseminated infection is rare, as most gonococci strains have limited ability to proliferate in the blood. However, certain strains can enter the circulation and cause disseminated infections. This can lead to fever, severe purulent arthritis, and the formation of small, solitary, dispersed pustules on the skin, which become erythematous due to capillary dilation or congestion. In some cases, necrosis may occur. Both men and women can experience disseminated infections, but women, especially during pregnancy and menstruation, are more prone to developing this condition [16].

Lab diagnosis

Urogenital gonorrhoea typically has an incubation period of 2-8 days [21]. Clinical signs of gonorrhoea have been found to vary significantly between males and females [22]. In males with gonococcal urethritis, at least 90% show dysuria and evident urethral discharge, making syndromic detection a cost-effective and time-saving technique in many settings. Gram staining can be helpful in assessing signs in males with symptomatic urethritis. However, laboratory-based diagnostic tests play a crucial role in diagnosing gonorrhoea in males without symptoms, females, and individuals with extragenital (pharyngeal and rectal) infections, which are often asymptomatic or present with nonspecific symptoms. Detecting gonorrhoea microbiologically can be taxing in some regions due to limited expertise in experimental screening and reliance on symptomatic approaches for empirical antibiotic therapy [23]. Gram-negative diplococci can be identified by microscopy in stained smears, *N. gonorrhoeae *culture, and nucleic acid amplification tests (NAATs) that detect *N. gonorrhoeae* RNA or DNA [24].

Microscopy

In places with resource-limited settings, light microscopy of Gram-stained samples is sometimes the only method to presumptively identify *N. gonorrhoeae* infection. The Gram stain's sensitivity and specificity screen for the presence of typical diplococci, which are Gram-negative inside PMNLs (polymorphonuclear leukocytes). The sensitivity and specificity of the Gram stain can vary depending on the sample material [25]. Methylene Blue is an alternative stain that has demonstrated similarly high specificity and sensitivity for diagnosing urethritis in males [26].

Culture

Endocervical culture has a sensitivity of 80-90%. Selective media such as Thayer Martin medium (chocolate agar with added antibiotics) are mostly preferred for cervical swabs since they tend to include specific microorganisms. In suspected disseminated gonococcal infection (DGI), blood cultures (using automated blood culture equipment) and synovial fluid cultures should be performed.

Identification

It is crucial to differentiate gonococci from other commensal *Neisseria *species. Gonococci are found to produce catalase and oxidase and exclusively ferment glucose but not maltose or sucrose. MALDI-TOF and other automated technologies can be used for identification.

NAATs

Most high-income countries now recommend NAATs for gonorrhoea diagnosis. NAATs have become the primary diagnostic test because they allow non-invasive specimen collection (self-collected swabs or urine, particularly vaginal swabs). This method does not require live cultures, enabling more accessible storage and transportation [27]. NAATs generally show high specificity with superior sensitivity (varies depending on the anatomical site tested and the specific NAAT used) compared to culture. They offer faster results (many later-generation NAAT platforms allow for high automation and throughput) and can simultaneously detect other STD-associated bacteria, especially *Chlamydia trachomatis* [28]. Initially, various PCR-based NAATs developed in-house were used locally and are still employed for diagnosis in resource-limited settings or as confirmatory tests [29]. In-house NAATs often target conserved gene regions such as the Gyra (encoding DNA subunit A gyrase), porA pseudogene, cppB (encoding B protein cryptic plasmid), opa gene, and *N. gonorrhoeae* methyl-transferase genes. Few studies have compared the performance of in-house NAATs to commercially available and FDA-regulated NAATs [30]. In high-income countries, in-house NAATs have mostly been replaced by commercial NAATs that are thoroughly validated and have obtained FDA regulatory clearance [31].

Point-of-care tests (POCTs)

Developing reliable and rapid point-of-care diagnostics (POCT) for gonorrhoea is a significant goal [32]. POCTs may provide precise and rapid detection to enable specific therapy in situations where it is currently not feasible, such as when only syndromic care is available, patients are unlikely to return for treatment, or when screening individuals who do not exhibit symptoms [33]. Ideally, POCTs should meet the 'ASSURED' characteristics, being affordable, user-friendly, specific, sensitive, durable, fast, and equipment-free (or requiring minimal equipment powered by solar or batteries) [34]. POCTs refer to any diagnostic test that provides accurate and rapid test results in a single clinical visit (Table 1). Gram stain is a well-known example of a POCT [35].

**Table 1:** Diagnostic tests for Neisseria gonorrhoeae NAATs: nucleic acid amplification tests

Parameter	Microscopy	Culture	NAATs
Specimen Type			
Urine	No	No	No
Urethral swab	Yes	Yes	Yes
Rectal swab	No	Yes	No
Pharyngeal swab	No	Yes	No
Conjunctival swab	Yes	Yes	Yes

Prophylaxis

Currently, there is no specific vaccination available for gonococci infection. Standard preventive measures should be taken, which include early detection of cases, health education promoting safe sex practices such as condom use, contact tracing, and treatment of both partners.

Complications

Gonorrhoea-related complications can have substantial socioeconomic consequences and may lead to fundamental morbidity. Non-detection or inadequate treatment can exacerbate the condition, resulting in severe reproductive health complications, including infertility, PID, chronic pelvic pain, and ectopic pregnancy [18]. In rare cases of disseminated gonococcal infection, it may manifest as endocarditis or septic arthritis. Gonorrhoeal conditions can also cause inflammation of the liver capsule and Fitz-Hugh-Curtis syndrome, leading to male infertility and intraabdominal adhesions in females. Certain complications are specific to men, such as prostatitis, proctitis, and epididymitis [19]. Additionally, newborns can contract the infection through eye contact with genital secretions, leading to significant obstetrical delivery difficulties, including conjunctivitis resulting in blindness. Gonorrhoea infection also increases the risk of HIV-AIDS transmission through sexual contact [36].

Antimicrobial resistance generally manifests in *N. gonorrhoeae*, the infectious agent of the disease, which correlates with local, regional, and national antibiotic use. Some countries, like Holland, have observed a lower incidence of gonococcal resistance due to reduced utilisation of cephalosporins, macrolides, fluoroquinolones, and other antibiotics compared to nations with high antibiotic consumption [37].

## Conclusions

*N. gonorrhoeae* is a sexually transmitted disease that is rapidly spreading worldwide and poses numerous serious health risks. Despite the development of several vaccines and antibiotics targeting this bacterium, its increasing resistance has become a concern. Significant progress has been made in controlling the spread of *N. gonorrhoeae*, but there remains a substantial amount of work to be done to effectively counter the threat it poses to global health.
